# Synergistic Activity of Niclosamide in Combination With Colistin Against Colistin-Susceptible and Colistin-Resistant *Acinetobacter baumannii* and *Klebsiella pneumoniae*

**DOI:** 10.3389/fcimb.2018.00348

**Published:** 2018-10-03

**Authors:** Rafael Ayerbe-Algaba, María Luisa Gil-Marqués, Manuel Enrique Jiménez-Mejías, Viviana Sánchez-Encinales, Raquel Parra-Millán, María Eugenia Pachón-Ibáñez, Jerónimo Pachón, Younes Smani

**Affiliations:** ^1^Clinic Unit of Infectious Diseases, Microbiology and Preventive Medicine, Institute of Biomedicine of Seville (IBiS), University Hospital Virgen del Rocío/CSIC/University of Seville, Seville, Spain; ^2^Department of Medicine, University of Seville, Seville, Spain

**Keywords:** repurposing drug, niclosamide, colistin, synergistic effect, *Acinetobacter baumannii*, *Klebsiella pneumoniae*

## Abstract

Colistin is among the few antibiotics effective against multidrug-resistant *Acinetobacter baumannii* and *Klebsiella pneumoniae* clinical isolates. However, in the last few years, colistin-resistant *A. baumannii* and *K. pneumoniae* strains have emerged. Therefore, combination therapies, between colistin and other old drugs, restoring the activity of colistin are required. The main objective of this study was to analyse the activity of niclosamide, an anthelmintic drug, in combination with colistin against colistin-susceptible (Col-S) and colistin-resistant (Col-R) *A. baumannii* and *K. pneumoniae*. The MIC were determined by microdilution assay and the time-kill curves were performed. The zeta potential of Col-S and Col-R of *A. baumannii* and *K. pneumoniae* in presence of niclosamide was assessed. Niclosamide in combination with colistin showed improved activity against Col-S and Col-R *A. baumannii* and *K. pneumoniae*. Time-killing curves showed synergic activity between niclosamide and colistin against Col-S and Col-R *A. baumannii* and *K. pneumoniae*, especially when niclosamide or colistin was added for second time at 4 h of the 24 h killing curve. Col-R *A. baumannii* and *K. pneumoniae* in presence of niclosamide exhibited a greater negative charge (−34.95 ± 0.35 mV and −38.85 ± 0.92 mV; *P* < 0.05) than Col-R *A. baumannii* and *K. pneumoniae* in absence of niclosamide (−26.85 ± 3.65 mV and −35.27 ± 0.72 mV). These data suggest that niclosamide might be combined with colistin, being a potential alternative for treatment of Col-R Gram-negative bacilli infections.

## Introduction

A number of infections caused by multidrug-resistant *Acinetobacter baumannii* and *Klebsiella pneumoniae* required the use of colistin, but both pathogens may rapidly acquire specific resistance mechanisms against colistin (Bonnin et al., 2013; Ah et al., 2014). Nowadays, the rates of colistin resistance worldwide vary between 3 and 28% for *A. baumannii*, and 2.8 and 10.5% for *K. pneumoniae* (Fernández-Cuenca et al., 2013; Ah et al., 2014). In this context, combination therapies between colistin and other drugs are among the new promising strategies to treat bacterial infections (Vila and Pachón, 2012; Cassir et al., 2014).

Classical combinations between colistin and other antimicrobial agents to which the isolate is resistant have been reported (Vila and Pachón, 2012; Paul et al., 2014). Their use has remained wide *in vitro* and in animal experimental model of infections (Vila and Pachón, 2012; Zusman et al., 2013). Few randomized controlled trials examining specific combinations have been completed or are ongoing, and are not sufficient to guide clinical practice (Paul et al., 2014; Poulikakos et al., 2014). This joins with the relatively short window of therapeutic application for severely ill patients for some combinations, and for the rapid emergence of drug resistance (Poulikakos et al., 2014).

In this context, “repurposing drugs,” defined as investigating new uses for already existing drugs, have gained renewed interest, as reflected by several recent studies (Chopra et al., 2010; Younis et al., 2015; Tharmalingam et al., 2018), and using them associated with colistin (Antunes et al., 2012; Zemke et al., 2014).

Niclosamide is an anthelmintic drug widely used for treating tape worm infection in humans and has lately been shown to possess anti-cancer and anti-diabetic activities (Tao et al., 2014; Ye et al., 2014). Niclosamide has also been identified as a potent anti-bacterial drug against *Helicobacter pylori* (Tharmalingam et al., 2018) and *Pseudomonas aeruginosa* by the inhibition of *quorum sensing* and various virulence genes, and by the reduction of elastase and pyocyanin levels (Imperi et al., 2013; Costabile et al., 2015). Moreover, Rajamuthiah et al., have reported that niclosamide present bacteriostatic activity against *Staphylococcus aureus* probably due the damage in their bacterial membrane but not against *A. baumannii, P. aeruginosa, E. coli*, and *K. pneumoniae* (Rajamuthiah et al., 2015; Gwisai et al., 2017). Currently there is no study regarding the combination between colistin and niclosamide to restore the activity of colistin against Gram-negative bacilli.

The aim of this study was to determine the *in vitro* activity of niclosamide in combination with colistin against colistin-susceptible and colistin-resistant *A. baumannii* and *K. pneumoniae* strains.

## Materials and methods

### Bacterial strains

Reference colistin-susceptible (Col-S) *A. baumannii* ATCC 17978 strain (Baumann et al., 1968), and 13 clinical colistin-resistant (Col-R) *A. baumannii* strains isolated from an hospital outbreak in 2000 in Spain (Valencia et al., 2009) were used in this study. We also used reference Col-S *K. pneumoniae* CECT 997 strain (Reading and Cole, 1977), one Col-S and 2 Col-R clinical *K. pneumoniae* strains (Pachón-Ibáñez et al., 2018).

### *Pmra, pmrb, mgrb, phop*, and *phoq* genes amplification and sequencing

In order to investigate the possible contribution of *pmrAB, mgrB*, and *phoPQ* to the colistin resistance in *A. baumannii* and *K. pneumoniae*, these genes were analyzed to detect any genetic alteration. DNA samples were obtained from the isolates by heating the colonies in water at 96°C. The genes were amplified using the primers listed in Table 1. The obtained bands were purified with the kit MEGAquick-spin plus (iNtRON Biotechnology, USA) and sequenced at the Institute of Biomedicine of Seville. The nucleotide and deduced protein sequences were analyzed using the Serial Cloner program (http://serialbasics.free.fr/Serial_Cloner.html).

**Table 1:** Primers list used in this study.

**Pathogen**	**Gene**	**Primer name**	**Sequence**	**Amplicon size (bp)**	**References**
*A. baumannii*	*pmrAB*	pmrAB-F	ATGACAAAAATCTTGATGAT	1,335	López-Rojas et al., 2011
		pmrAB-R	TCACGCTCTTGTTTCATGTA		López-Rojas et al., 2011
*K. pneumoniae*	*pmrAB*	pmrA-F	CGCAGGATAATCTGTTCTCCA	808	Haeili et al., 2017
		pmrA-R	GGTCCAGGTTTCAGTTGCAA		Haeili et al., 2017
		pmrB-F1	GCGAAAAGATTGGCAAATCG	659	Haeili et al., 2017
		pmrB-R1	GGAAATGCTGGTGGTCATCTGA		Haeili et al., 2017
		pmrB-F2	CCCTGAATCAGTTGGTTTC	714	Haeili et al., 2017
		pmrB-R2	ATCAATGGGTGCTGACGTT		Haeili et al., 2017
	*mgrB*	mgrB-extF	AAGGCGTTCATTCTACCACC	253	Poirel et al., 2015
		mgrB-extR	TTAAGAAGGCCGTGCTATCC		Poirel et al., 2015
	*phoPQ*	phoP-F	GAGCGTCAGACTACTATCGA	912	Haeili et al., 2017
		phoP-R	GTTTTCCCATCTCGCCAGCA		Haeili et al., 2017
		phoQ-F1	CCACAGGACGTCATCACCA	636	Haeili et al., 2017
		phoQ-R1	AGCTCCACACCATATAGCTG		This study
		phoQ-F2	GAACAGGGCGACGACTCTG	617	This study
		phoQ-R2	TGAGAGCGGAAGTCAGGCT		This study
		phoQ-F3	GATGCTGGAGCAGATAAGCC	621	This study
		phoQ-R3	GCAGGTGTCTGACAGGGATT		This study

### *in vitro* susceptibility testing

MIC of colistin (Sigma, Spain), MIC of niclosamide (Sigma, Spain), and MIC of colistin in presence of different concentrations of niclosamide (between 0.5 and 4 μM) against Col-S and Col-R references and clinical *A. baumannii* and *K. pneumoniae* strains were determined in two independent experiments by broth microdilution assay according to CLSI recommendations for *A. baumannii* and EUCAST recommendations for *K. pneumoniae* (Clinical and Laboratory Standards Institute, 2016 European Committee on Antimicrobial Susceptibility European Committee on Antimicrobial Susceptibility Testing [EUCAST], 2016). The initial inoculum of 5 x 10^5^ CFU/mL for each strain was used in microtiter plates V (Deltlab, Spain) in presence of colistin, niclosamide, or colistin plus niclosamide, and incubated for 16–18 h at 37°C. *Escherichia coli* ATCC 25922 was used as control strain.

### Time-kill kinetic assays

Time-kill curves of *A. baumannii* ATCC 17978 and Col-R #11 strains and *K. pneumoniae* CECT 997 and KPc21 strains with starting inoculum of 1 × 10^6^ CFU/mL, conducted on Mueller Hinton broth cation-adjusted, in presence of 2 μM niclosamide and colistin (sub-MIC) alone or in combination were performed in two independent experiments as previously described (Smani et al., 2011).

Moreover, in some conditions niclosamide was added for a second time 4 h after bacterial inoculation in order to avoid the antagonism effect of niclosamide with colistin observed in the first 4 h. In the same way colistin was added for a second time 4 h after bacterial inoculation because the half-life of colistin in bacterial culture broth is 4 h (Owen et al., 2007; Bergen et al., 2010). Bacterial cultures without drugs were carried out in parallel as controls. Tubes of each condition were incubated at 37°C with shaking and samples were taken at 0, 2, 4, 8, and 24 h and serially diluted. Viable counts were determined by plating 100 μL of control, test cultures, or dilutions at the indicated times onto sheep blood agar plates (Beckton Dickinson, USA). Plates were incubated for 24 h, and after colony counts, the log of viable cells (CFU/mL) was determined. Synergy was defined as a reduction ≥ 2 log CFU/mL with the combination respect to the more active drug (Pachón-Ibáñez et al., 2018). Thus, niclosamide was considered synergistic when in combination with colistin reduced the bacterial concentration ≥ 2 log CFU/mL with respect to colistin alone.

### Zeta potential measurements

Zeta potential measurements were performed as previously described with minor modifications (Soon et al., 2011). Briefly, the bacterial surface was cleansed by washing twice with Milli-Q water, resuspended in Milli-Q water at 10^8^ CFU/mL, and diluted 10-fold in the same medium immediately prior to zeta potential measurement. The resulting suspensions were used to fill clear disposable folded capillary zeta cells (Malvern, UK).

To examine the effect of niclosamide treatment on Col-S and Col-R *A. baumannii* and *K. pneumoniae* strains, 2 μM niclosamide was added to 5 mL of bacterial culture at 10^8^ CFU/mL, then incubated in a shaking bath (37°C, 180 rpm) for 20 min and prepared for zeta potential analysis as described above. The zeta potential measurement (mV) of bacterial cells was measured at 25°C with a zeta potential analyzer at 150 V (Zetasizer Nano ZS; Malvern Instruments, Malvern, UK).

### Statistical analysis

Group data are presented as mean ± SEM. Student *t*-test was used to determine differences between means. Differences were considered significant at *P* < 0.05. The SPSS (version 17.0) statistical package was used (SPSS Inc.).

## Results

### Colistin MIC and resistance mechanisms

The MIC for the Col-R *A. baumannii* and *K. pneumoniae* strains ranged from 32 to > 256 μg/mL, while those for the susceptible reference strains were 0.5 μg/mL. The analysis of the *pmrA, pmrB, mgrB, phoP*, and *phoQ* sequences showed that Col-R *A. baumannii* strains presented only different amino acids substitution in *pmrB* (López-Rojas et al., 2016). Col-R *K. pneumoniae* strains presented IS1 transposase insertion in *mgrB* or different amino acids substitution in *pmrA* and *pmrB*, without substituting amino acid in *phoP* and *phoQ* (Table 2).

**Table 2:** Colistin MICs and description of Col-S and Col-R *A. baumannii* and *K. pneumoniae* strains used in this study.

**Pathogen**	**Strain**	**Reference/Source**	**Description**	**Colistin MIC (μg/ml)**
*A. baumannii*	ATCC 17978	Baumann et al., 1968	Colistin-susceptible reference strain	0.5
	#1	Valencia et al., 2009; López-Rojas et al., 2016	PDR clinical isolate with A236E amino acid substitution of *pmrB*	256
	#10	Valencia et al., 2009; López-Rojas et al., 2016	PDR clinical isolate with F387Y and S403F amino acids substitution of *pmrB*	>256
	#11	Valencia et al., 2009; López-Rojas et al., 2016	PDR clinical isolate with R263C amino acid substitution of *pmrB*	256
	#14	Valencia et al., 2009; López-Rojas et al., 2016	PDR clinical isolate with R263C amino acid substitution of *pmrB*	256
	#16	Valencia et al., 2009; López-Rojas et al., 2016	PDR clinical isolate with S403F amino acid substitution of *pmrB*	>256
	#17	Valencia et al., 2009; López-Rojas et al., 2016	PDR clinical isolate with R263C amino acid substitution of *pmrB*	64
	#19	Valencia et al., 2009; López-Rojas et al., 2016	PDR clinical isolate with T13A and S17G amino acids substitution of *pmrB*	>256
	#20	Valencia et al., 2009; López-Rojas et al., 2016	PDR clinical isolate with A227V and M308T amino acids substitution of *pmrB*	>256
	#21	Valencia et al., 2009; López-Rojas et al., 2016	PDR clinical isolate with P170Q amino acid substitution of *pmrB*	256
	#22	Valencia et al., 2009; López-Rojas et al., 2016	PDR clinical isolate with P170Q amino acid substitution of *pmrB*	>256
	#24	Valencia et al., 2009; López-Rojas et al., 2016	PDR clinical isolate with R263C amino acid substitution of *pmrB*	64
	#99	Valencia et al., 2009; López-Rojas et al., 2016	PDR clinical isolate with A227V amino acid substitution of *pmrB*	>256
	#113	Valencia et al., 2009; López-Rojas et al., 2016	PDR clinical isolate with L5S, R207C and G426S amino acids substitution of *pmrB*	>256
*K. pneumoniae*	CECT 997	Reading and Cole, 1977	Colistin-susceptible reference strain	0.5
	KPc07	Pachón-Ibáñez et al., 2018	Colistin-susceptible clinical isolate	0.5
	KPc21	Pachón-Ibáñez et al., 2018	Clinical isolate containing IS1 transposase insertion at nucleotide 22 of *mgrB*	64
	KPc29	Pachón-Ibáñez et al., 2018	Clinical isolate with a G56E and M215I amino acids substitution of *pmrA*, and R63P; S68N and R255G amino acids substitution of *pmrB*	32

### *in vitro* activity of niclosamide in combination with colistin against col-S and col-R *a. baumannii* and *k. pneumoniae*

Niclosamide alone or in combination with colistin was tested against reference and clinical Col-S and Col-R *A. baumannii* and *K. pneumonia*e strains. The MIC is shown in Table 3. Niclosamide alone showed a range of MIC from 6.25 to 400 μM for Col-S and Col-R *A. baumannii* strains, and from 400 to >800 μM for Col-S and Col-R *K. pneumoniae* strains.

**Table 3:** Determination of MIC of niclosamide and colistin alone or in combination against Col-S and Col-R *A. baumannii* and *K. pneumoniae*.

**Pathogen**	**Strain**	**Nicl MIC (μM)**	**Col MIC (μg/ml)**	**Col MIC (**μ**g/ml)**			
				**+ Nicl 4 μM**	**+ Nicl 2 μM**	**+ Nicl 1 μM**	**+ Nicl 0.5 μM**
*A. baumannii*	ATCC 17978	25	0.5	< 0.03	< 0.03	< 0.03	0.25
	#1	25	256	**0.06**	**0.5**	256	>256
	#10	400	>256	**0.03**	**0.5**	32	ND
	#11	200	256	**0.06**	**0.25**	32	>256
	#14	6.25	256	**0.06**	**0.25**	32	256
	#16	50	>256	**0.03**	**0.5**	64	ND
	#17	12.5	64	<**0.03**	**0.125**	8	64
	#19	12.5	>256	**0.125**	**2**	128	>256
	#20	12.5	>256	**0.125**	**1**	64	>256
	#21	200	256	<**0.03**	<**0.03**	8	16
	#22	200	>256	<**0.03**	<**0.03**	**0.5**	16
	#24	64	64	**0.06**	**0.125**	8	64
	#99	400	>256	**0.03**	**0.5**	16	>256
	#113	400	>256	**0.06**	**1**	8	256
*K. pneumoniae*	CECT 997	>800	0.5	**0.06**	**0.06**	**0.125**	**0.125**
	KPc07	400	0.5	**0.06**	**0.06**	**0.125**	**0.125**
	KPc21	>800	64	<**0.015**	<**0.015**	**0.15**	32
	KPc29	800	32	**0.25**	**0.5**	**1**	4

*Values in bold indicate the condition in which the presence of niclosamide changed the bacterial colistin susceptibility from resistant to susceptible. One micromolar of niclosamide correspond to 0.33 μg/ml. Nicl, niclosamide; Col, colistin; ND, not determined*.

Niclosamide at 1, 2, and 4 μM in combination with colistin increased significantly the activity of colistin against all Col-S and Col-R strains. In contrast niclosamide, at 0.5 μM in combination with colistin didn't increased the activity of colistin against most of Col-R *A. baumannii*. For the rest of experiments, we chose 2 μM as niclosamide optimal concentration.

### Time-killing curves

**Figure 1:**
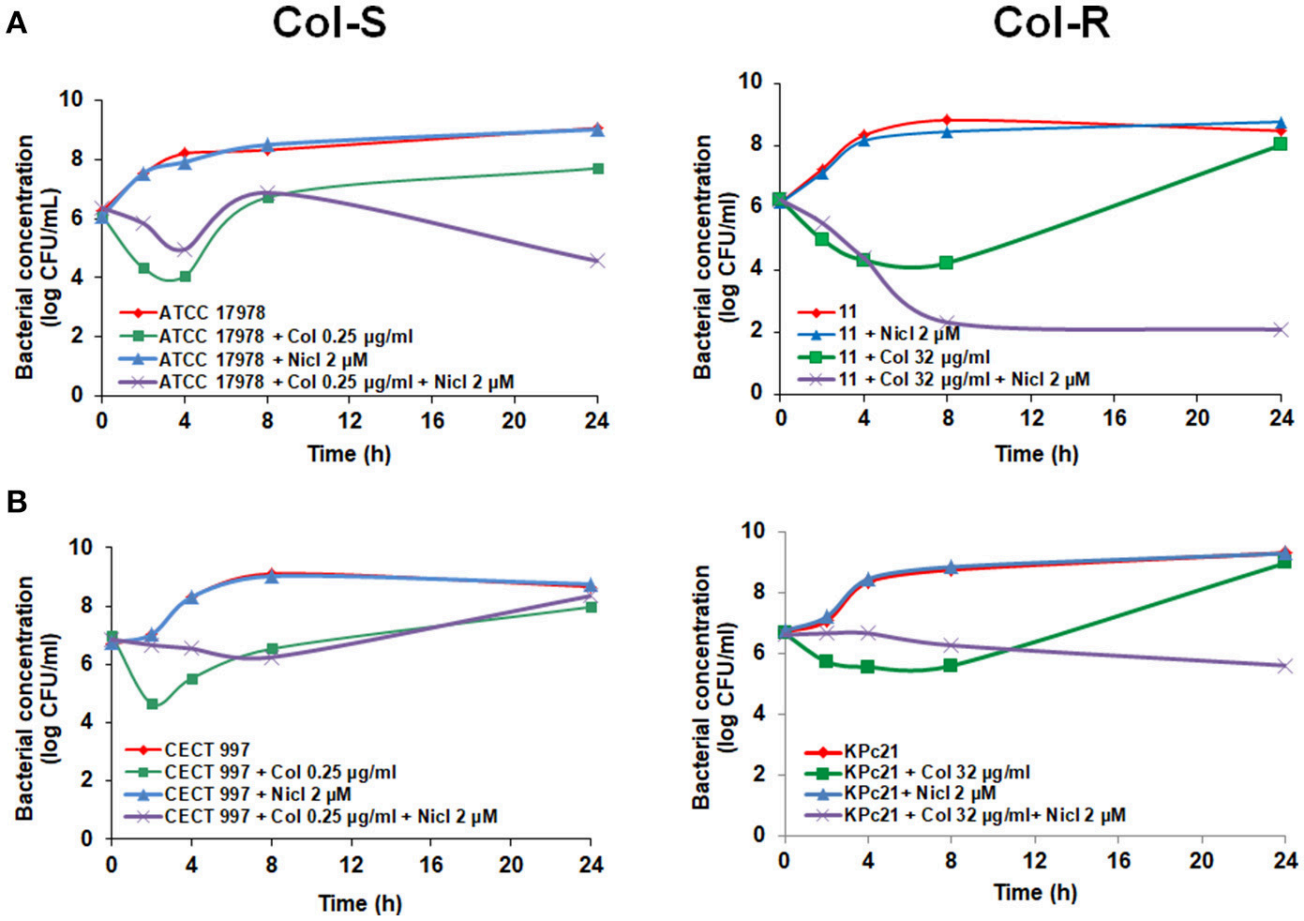
Early addition of niclosamide potentiates the colistin activity against Col-S and Col-R *A. baumannii* and *K. pneumoniae* strains. Time-kill curves of *A. baumannii* ATCC 17978 and #11 strains **(A)** and *K. pneumoniae* CECT 997 and KPc21 strains **(B)** in presence of niclosamide (0 or 2 μM), and colistin (sub-MIC) alone or in combination with niclosamide for 24 h. 1 μM of niclosamide correspond to 0.33 μg/ml. Nicl, niclosamide, Col, colistin.

We examined the ability of niclosamide in combination with colistin to kill Col-S and Col-R *A. baumannii* strains (ATCC 17978 and #11) and Col-S and Col-R *K. pneumoniae* strains (CECT 997 and KPc21) in time course assays. Two micromolar niclosamide in combination with 0.25 μg/mL colistin, a colistin sub-MIC of ATCC 17978 strain, showed in the first 4 h no synergistic activity with respect to colistin alone, followed by later synergistic activity decreasing the bacterial cell count by 3.14 log CFU/mL with respect to colistin alone at 24 h (Figure 1A). Combination of 2 μM niclosamide with 8 μg/mL colistin, a colistin sub-MIC of #11 strain, showed higher synergistic activity, decreasing the bacterial cell count with respect to colistin alone by 5.93 log CFU/mL at 24 h (Figure 1A).

In the case of *K. pneumoniae*, 2 μM niclosamide in combination with 0.25 μg/mL colistin, a colistin sub-MIC of CECT 997 strain, didn't showed synergistic effect with colistin during 24 h (Figure 1B). In contrast, 2 μM niclosamide in combination with 32 μg/mL colistin, a colistin sub-MIC of KPc21 strain, showed synergistic activity with respect to colistin alone decreasing the bacterial cell count by 3.38 log CFU/mL at 24 h (Figure 1B). In a control experiment, 2 μM niclosamide had no effect on the growth of Col-S and Col-R *A. baumannii* and *K. pneumoniae* strains (Figure 1A,B).

With these results, since the half-life of colistin in bacterial culture broth is 4 h (Owen et al., 2007; Bergen et al., 2010) and can be degraded during time-kill curve experiments (Mohamed et al., 2014), we cannot rule out the possibility that the absence of synergistic activity of niclosamide in combination with colistin observed especially with *K. pneumoniae* CECT 997 strain (Figure 1B) would be due to colistin degradation in the bacterial culture broth.

**Figure 2:**
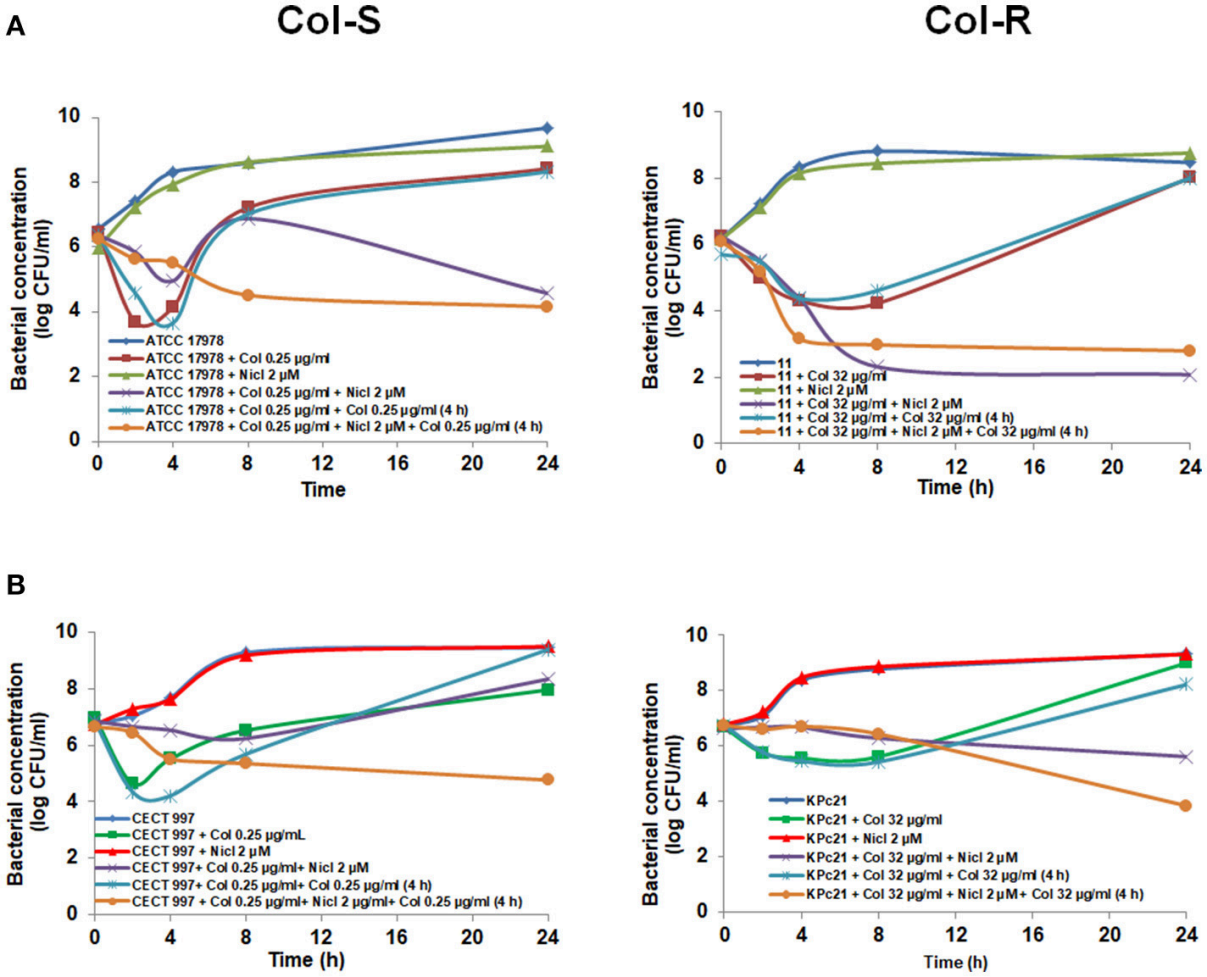
Niclosamide potentiates the colistin activity against Col-S and Col-R *A. baumannii* and *K. pneumoniae* strains after second time addition of colistin. Time-kill curves of *A. baumannii* ATCC 17978 and #11 strains **(A)**, *K. pneumoniae* CECT 997 and KPc21 strains **(B)**, in presence of 2 μM niclosamide and colistin (sub-MIC) alone, or in combination with or without addition of colistin for second time 4 h after bacterial addition. One micromolar of niclosamide correspond to 0.33 μg/ml. Nicl, niclosamide; Col, colistin.

Consequently, to maintain the colistin concentration in the medium, in the following experiments we added 0.25 μg/mL colistin after 4 h post-incubation with the initial 2 μM niclosamide and 0.25 μg/mL colistin, or with 0.25 μg/mL colistin alone. This approach produced synergy between niclosamide and colistin, decreasing the bacterial cell count of CECT 997 strain by 4.62 log CFU/mL with respect to colistin plus colistin at 24 h (Figure 2B). Similarly, the addition of 32 μg/mL colistin after 4 h post-incubation with niclosamide and 32 μg/mL colistin decreased the growth of KPc21 strain by 4.38 log CFU/mL with respect to colistin plus colistin at 24 h (Figure 2B). In the case of *A. baumannii* ATCC 17978 strain, the addition of colistin for second time at 4 h increased the synergy between niclosamide and colistin plus colistin decreasing the bacterial cell count by 2.37 log CFU/mL with respect to niclosamide plus colistin, without the addition of colistin at 4 h, at 8 h (Figure 2A). In contrast, with #11 strain the addition of colistin for second time did not improve the synergy between niclosamide and colistin observed in the (Figures 1, 2A).

**Figure 3:**
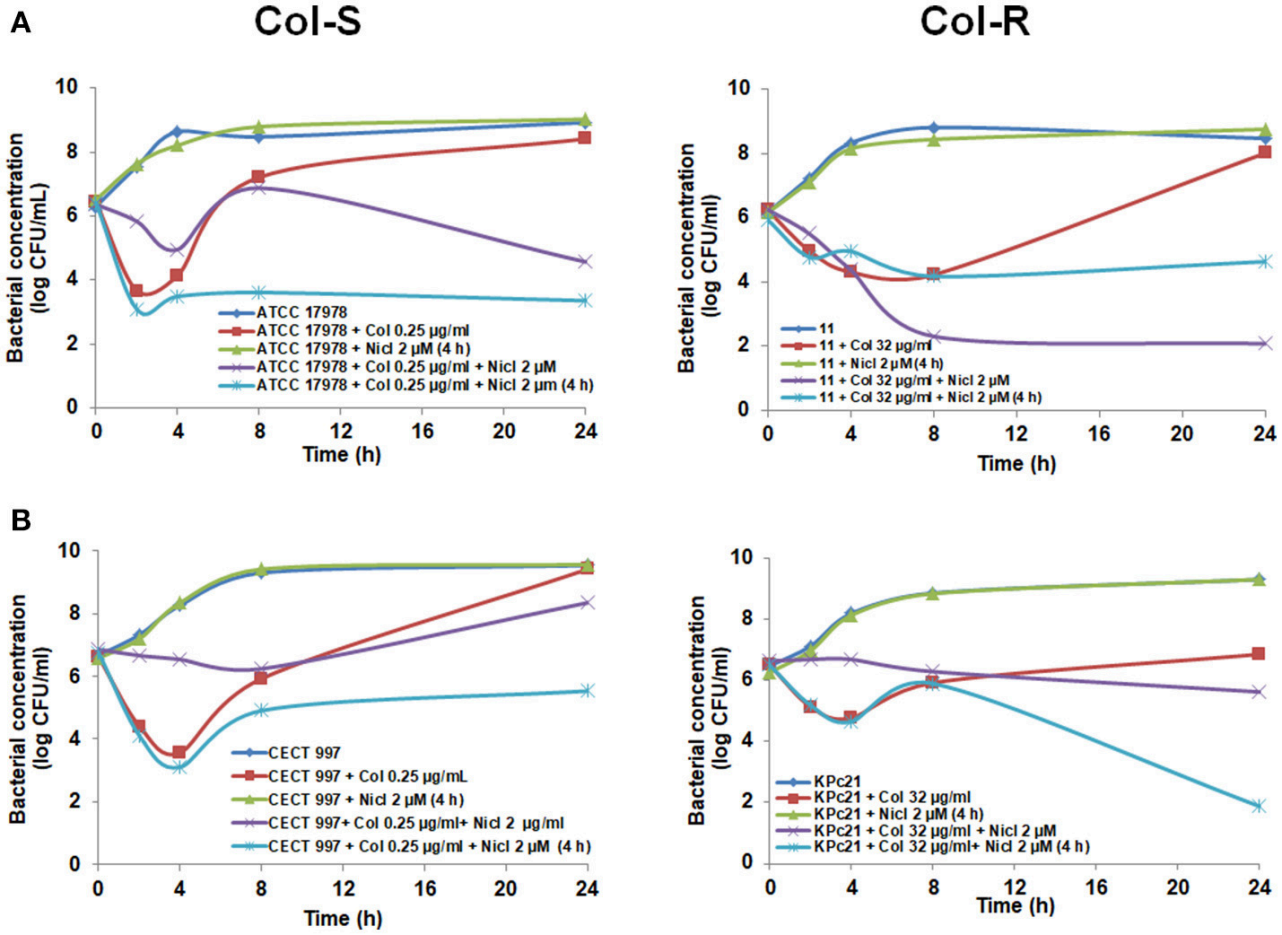
Late addition of niclosamide potentiates the colistin activity against Col-S and Col-R *A. baumannii* and *K. pneumoniae* strains. Time-kill curves of *A. baumannii* ATCC 17978 and #11 strains **(A)**, *K. pneumoniae* CECT 997 and KPc21 strains **(B)**, in presence of colistin (sub-MIC) alone with or without addition of 2 μM niclosamide 4 h after bacterial addition. One micromolar of niclosamide correspond to 0.33 μg/ml. Nicl, niclosamide; Col, colistin.

Furthermore, to avoid the antagonism effect of niclosamide with colistin observed in the first 4 h (Figures 1, 2), we added 2 μM niclosamide after 4 h post-incubation with 0.25 and 8 μg/mL colistin, sub-MIC of *A. baumannii* ATCC 17978 and #11 strains, respectively. We observed a decrease in the growth of ATCC 17978 and #11 strains by 5.06 and 3.38 log CFU/mL, respectively, with respect to colistin alone at 24 h (Figure 3A).

In the case of *K. pneumoniae*, we added 2 μM niclosamide after 4 h post-incubation with 0.25 and 32 μg/mL colistin, sub-MIC of CECT 997 and KPc21 strains, respectively. We observed a decrease in the growth of CECT 997 and KPc21 strain by 3.89 and 4.98 log CFU/mL, respectively, with respect to colistin alone at 24 h (Figure 3B). In the control experiment, addition of 2 μM niclosamide after 4 h post-bacterial incubation had no effect on the growth of Col-S and Col-R *A. baumannii* and *K. pneumoniae* strains (Figures 3A, 3B).

### Zeta potential

**Figure 4:**
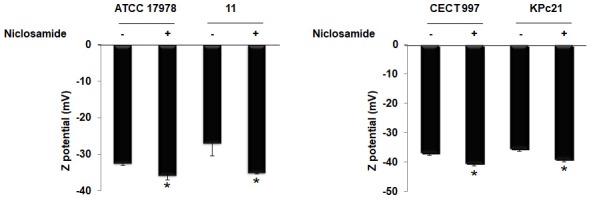
Zeta potential of Col-S and Col-R *A. baumannii* and *K. pneumoniae* strains in presence and absence of niclosamide. Data are the mean ± SEM. ^*^P < 0.05 untreated vs. treated with niclosamide.

Figure 4 illustrates the zeta potential of *A. baumannii* ATCC 17978 and #11 strains in presence and absence of niclosamide. Analysis of zeta potential revealed that treatment of ATCC 17978 and #11 strains with 2 μM niclosamide exhibited significantly high negative surface charge by −35.62 ± 1.32 mV and −34.95 ± 0.35 mV, respectively with respect to ATCC 17978 and #11 strains without treatment with niclosamide, −32.33 ± 0.63 and −26.85 ± 3.65 mV, respectively. Similarly, analysis of zeta potential revealed that treatment of *K. pneumoniae* CECT 997 and KPc21 strains with 2 μM niclosamide exhibited significantly high negative surface charge by −40.23 ± 0.88 mV and −38.85 ± 0.93 mV, respectively with respect to CECT 997 and KPc21 strains without treatment with niclosamide, −36.7 ± 0.88 and −35.27 ± 0.72 mV, respectively (Figure 4).

## Discussion

Colistin resistance, although uncommon, is increasingly being reported among clinical Gram-negative bacilli isolates, and an understanding of its impact on the activity of antimicrobials is now evolving (Olaitan et al., 2014). Modification of LPS is one of the colistin resistance mechanisms in Gram negative bacilli that result in the increase of positive surface charge of the bacterial outer membrane (Olaitan et al., 2014).

Niclosamide's ability to carrier proton has been previously used to investigate the potential of niclosamide for blocking the acidification of endosomes in eukaryotic cells (Jurgeit et al., 2012). Thus, the use of niclosamide to carrier the proton in the outer membrane of Col-R strains could be helpful to restore the activity of colistin against Gram negative bacilli.

In the present study, the combination of niclosamide with colistin potentiates the activity of colistin against Col-S and especially Col-R *A. baumannii* and *K. pneumoniae* strains at 24 h, despite of the antagonist effect of niclosamide on the antibacterial effect of colistin against these strains in the first 4 h. Similar results regarding the antagonism of the colistin effect were observed in the first 4–8 h when colistin was combined with doripenem or ertapenem against a clinical isolate of *K. pneumoniae* resistant to colistin, doripenem, and ertapenem (Hong et al., 2013), and with levofloxacin against a clinical isolate of *A. baumannii* resistant to levofloxacin (Safarika et al., 2015). In the present study, this antagonism has been corrected when niclosamide was added 4 h after bacterial and colistin incubation.

It is noteworthy to mention that the use of niclosamide alone did not affect the growth of Col-S and Col-R *A. baumannii* and *K. pneumoniae* strains, which is consistent with previously published data in which niclosamide was not effective against Gram-negative bacilli including *A. baumannii* and *K. pneumoniae* (Rajamuthiah et al., 2015). In addition, the synergy between niclosamide and colistin was increased when colistin was added for second time 4 h post-bacterial due to a possible compensation of colistin degradation in the broth culture (Owen et al., 2007; Bergen et al., 2010).

It is well known that niclosamide does not cause significant bacterial cell envelope damage in Gram-positive pathogens (Rajamuthiah et al., 2015), and Gram-negative bacilli may have intrinsic resistance to niclosamide due to their functional and structural characteristics (Blair et al., 2014). Interestingly, despite the fact that niclosamide does not appear to inhibit *A. baumannii* and *K. pneumoniae* growth, we demonstrated, for the first time, that niclosamide increased the negative surface charge of Col-S and Col-R *A. baumannii* and *K. pneumoniae* strains. This effect was higher with Col-R than with Col-S *A. baumannii* and *K. pneumoniae* strains. This fact would be due to the proportion of negative and positive surface charge in these strains. Indeed, we observed that Col-R strains contain less negative surface charges than Col-S strains. These data are consistent with previously published reports, showing less negative surface charges in Col-R *A. baumannii* and *K. pneumoniae* (Soon et al., 2011; Velkov et al., 2014). Recently, it was reported that salicylanilide analogs inhibit *Clostridioides difficile* growth via membrane depolarization by dissipation of the bacterial membrane potential (Gooyit and Janda, 2016).

Furthermore, we showed that Col-R *A. baumannii* strain did not improve the synergy with niclosamide while adding the second colistin dose. A explanation for this data would be that the binding of colistin to the cell wall of this strain is saturated after the first addition of colistin in medium. Indeed, this strain, in presence of niclosamide, has presented lower zeta potential level (−34.95 ± 0.35 mV) than with *K. pneumoniae* CECT 997 strain (−40.23 ± 0.88 mV), *K. pneumoniae* KPc21 strain (−38.85 ± 0.93 mV), and *A. baumannii* ATCC 17978 strain (−35.62 ± 1.32 mV) which suggest the colistin binding saturation.

We have not shown that the incubation of *A. baumannii* and *K. pneumoniae* with niclosamide affect their OMPs profiles (data not shown). Thus, further investigations, including the integrity of bacterial membrane by transmission electron microscopy, are necessary to better understand how niclosamide acts synergistically with colistin against Col-S and Col-R *A. baumannii* and *K. pneumoniae* strains.

Concerning future developments of niclosamide as potent synergic drug with colistin, niclosamide derivative or same structural class of niclosamides, i.e., the salicylanilide oxyclozanide, need to be evaluated in combination with colistin. Previous studies showed that oxyclozanide present same activities as niclosamide against *P. aeruginosa* and *S. aureus* (Imperi et al., 2013; Rajamuthiah et al., 2015). Furthermore, It should be a very interesting exercise to assay *in vitro* the anti-quorum sensing of niclosamide (Imperi et al., 2013; Costabile et al., 2015) in combination with colistin and *in vivo* the colistin niclosamide combination in an infection model like the silk worm *Galleria mellonella*.

## Conclusions

Niclosamide has potentiated the effect of colistin against Col-S and Col-R *A. baumannii* and *K. pneumoniae* strains. This effect might be due to the alteration of negative surface charge proportion in the outer membrane of these strains. The results of this study provide new insights into the use of niclosamide in combination with colistin to treat the infections by Gram negative bacilli.

## Author contributions

JP and YS conceived the study. RA-A, MG-M, VS-E, RP-M, and MP-I carried out the experiments. RA-A and YS analyzed the data. MJ-M, MP-I, and JP have reviewed the manuscript and the experiments. RA-A and YS wrote the manuscript. All authors read and approved the final manuscript.

### Conflict of interest statement

The authors declare that the research was conducted in the absence of any commercial or financial relationships that could be construed as a potential conflict of interest.
